# Isoform requirement of clustered protocadherin for preventing neuronal apoptosis and neonatal lethality

**DOI:** 10.1016/j.isci.2022.105766

**Published:** 2022-12-08

**Authors:** Hiroaki Kobayashi, Kenji Takemoto, Makoto Sanbo, Masumi Hirabayashi, Takahiro Hirabayashi, Teruyoshi Hirayama, Hiroshi Kiyonari, Takaya Abe, Takeshi Yagi

**Affiliations:** 1 KOKORO-Biology Group, Graduate School of Frontier Biosciences, Osaka University, Suita 565-0871, Japan; 2 Division of Biophysical Engineering, Department of Systems Science, School of Engineering Science, Osaka University, Toyonaka 565-8531, Japan; 3 Section of Mammalian Transgenesis, Center for Genetic Analysis of Behavior, National Institute for Physiological Sciences, Okazaki 444-8585, Japan; 4 Department of Anatomy and Developmental Neurobiology, Tokushima University, Graduate School of Medical Science, Tokushima 770-8503, Japan; 5 Laboratory for Animal Resources and Genetic Engineering, RIKEN Center for Biosystems Dynamics Research, Kobe 6500047, Japan

**Keywords:** Cell biology, Developmental genetics, Neuroscience

## Abstract

Clustered protocadherin is a family of cell-surface recognition molecules implicated in neuronal connectivity that has a diverse isoform repertoire and homophilic binding specificity. Mice have 58 isoforms, encoded by *Pcdhα*, *β*, and *γ* gene clusters, and mutant mice lacking all isoforms died after birth, displaying massive neuronal apoptosis and synapse loss. The current hypothesis is that the three specific γC-type isoforms, especially γC4, are essential for the phenotype, raising the question about the necessity of isoform diversity. We generated *TC* mutant mice that expressed the three γC-type isoforms but lacked all the other 55 isoforms. The *TC* mutants died immediately after birth, showing massive neuronal death, and *γC3* or *γC4* expression did not prevent apoptosis. Restoring the *α*- and *β*-clusters with the three *γC* alleles rescued the phenotype, suggesting that along with the three γC-type isoforms, other isoforms are also required for the survival of neurons and individual mice.

## Highlights

• 55 cPcdh isoforms other than three *γ*C were necessary for neonatal survival
• *TC* mutants lacking 55 cPcdh isoforms except three *γ*C exhibited massive apoptosis
• Apoptosis-susceptive regions expressed *γ*C4 and stochastic isoforms combinatorially
• The expression of *γ*C3 or *γ*C4 did not prevent cells from apoptosis in *TC* mutants

## Graphical abstract

**Figure:**
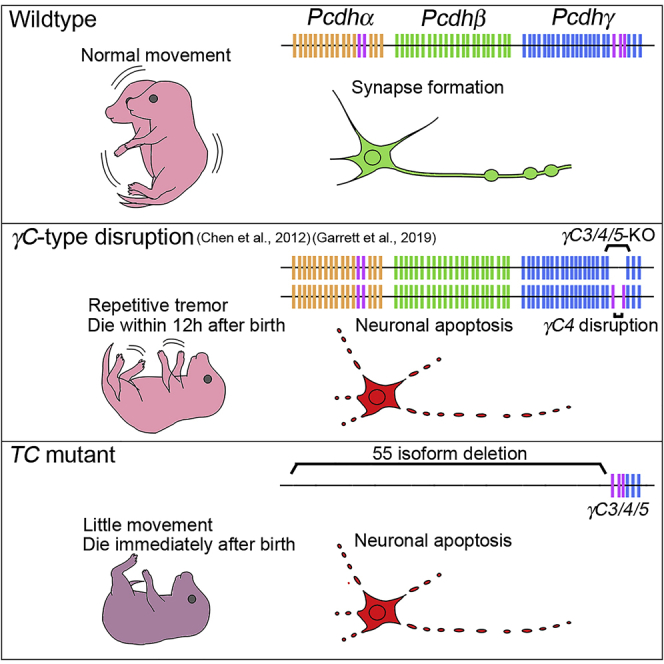

## Introduction

The rule governing the connectivity of a neural circuit is pivotal in constructing the brain, and the diversity of cell-surface recognition molecules has been implicated in the regulation of this connectivity. The nervous systems of insects and vertebrates have independently evolved different types of cell-surface recognition molecules with extraordinarily diverse isoforms, namely, Dscam1 in insects^1^ and clustered protocadherins in vertebrates (cPcdh).^2^^,^^3^^,^^4^ This suggests that the utilization of the isoform diversity, not the protein species itself, is essential in constructing the brain. Mice have 58 cPcdh isoforms encoded by three gene clusters, namely, *Pcdhα* (14 isoforms), *Pcdhβ* (22 isoforms), and *Pcdhγ* (22 isoforms).^5^^,^^6^ Individual neurons express a distinct combination of cPcdh isoform subsets in a stochastic manner.^7^^,^^8^^,^^9^^,^^10^ cPcdh proteins form *cis*-dimers promiscuously, with preferences for heterologous dimers with other isoforms that increase the variety of recognition units.^11^^,^^12^^,^^13^ cPcdh isoforms then interact strictly homophilically in *trans* at the cell surface of opposing neurons, such as at synapses, creating interaction specificity.^12^^,^^13^^,^^14^^,^^15^^,^^16^^,^^17^ Therefore, cPcdh can create cell-surface identity for cell recognition, which leads to the hypothesis that cPcdh works as a synaptic partner-selection molecule. However, this has not been proven at the synapse level yet.

Gene knockout studies targeting each of the three *cPcdh* clusters have shown that cPcdh plays a role in multiple aspects of recognition events, including axonal projection, dendritic self-avoidance, dendritic arbor complexity, and synapse formation.^18^^,^^19^^,^^20^^,^^21^^,^^22^^,^^23^^,^^24^^,^^25^^,^^26^^,^^27^^,^^28^^,^^29^^,^^30^^,^^31^ Mice lacking all 58 isoforms (*Δαβγ* mice) exhibit the most severe phenotype. They die immediately after birth due to massive neuronal death and synaptic loss in the brainstem and spinal cord. Genetically blocking apoptosis in *Δαβγ* mice by deleting the *Bax* gene cannot rescue neonatal lethality, synaptic defects, or neural circuit malfunction, suggesting that *Δαβγ* mice have an abnormally wired neural network.^32^ A similar neonatal lethal phenotype is also observed in *Δβγ* and *Δγ* mice, whereas *Δα*, *Δβ*, and *Δαβ* mice can survive, suggesting that *Pcdhγ* plays a dominant role in neural network formation. However, since the phenotypic severity of neuronal death and synaptic loss increases with the number of deleted clusters (*Δγ*<*Δβγ*<*Δαβγ*), all three *Pcdh* clusters may cooperatively contribute to neuronal survival and functional neural circuit formation.^22^

Among the 58 isoforms, the last two isoforms in the *Pcdhα* cluster and the last three isoforms in the *Pcdhγ* cluster are distinctly categorized as C-type isoforms based on sequence homology.^4^ The C-type isoforms are more ubiquitously expressed (although not in all neurons) compared with the other 53 variable isoforms that are stochastically and combinatorially expressed in individual neurons.^8^^,^^9^^,^^10^^,^^18^^,^^28^ Interestingly, the triple γC-type isoform knockout (*TCKO*), which lacks *γC3*, *γC4*, and *γC5* isoforms, exhibits a phenotype similar to the *Pcdhγ* null mutant, whereas the triple γA-type isoform knockout, which lacks *γA1*, *γA2*, and *γA3* isoforms, shows no discernible abnormalities.^33^^,^^34^ Subsequently, it was shown that *γC4* is the only responsible and sufficient isoform for the survival of both neurons and individual mice.^35^ CRISPR/Cas9-mediated disruption of *γC4* caused neuronal apoptosis and neonatal lethality in mice, whereas the disruption of all the other *γ* isoforms except *γC4* resulted in a grossly normal phenotype.^35^ This raised the question about the role and the necessity of the other 55 isoforms. Thus, in this study, we aimed to generate mutant mice that only express the three γC-type isoforms (*TC* for triple γC-type) but lacked all the other 55 isoforms including *Pcdh α* and *β*.

*TC* mutants died immediately following birth and exhibited massive apoptotic cell death in a specific brain region of the basal forebrain and in the large area of the brainstem that contains the reticular formation. On the other hand, *αβ/TC* compound mutants that carry the *TC* allele complemented with *α* and *β* alleles survived to adulthood, indicating that the three γC-type isoforms in the *TC* allele were functional for individual survival. *In situ* hybridization (ISH) showed that the wild-type brain regions where apoptotic cell death occurred in *TC* mutants expressed both *γC3* and *γC4* isoforms and the stochastic isoforms from the *Pcdhα*, *β*, and *γ* clusters in combination. This result suggests that the essential *γC4* isoform is always expressed with the other stochastic isoforms, and this combination is necessary for the survival of neurons and individual mice.

## Results

### *TC* mutant neonates die at birth despite the expression of the three γC-type isoforms

**Figure 1:**
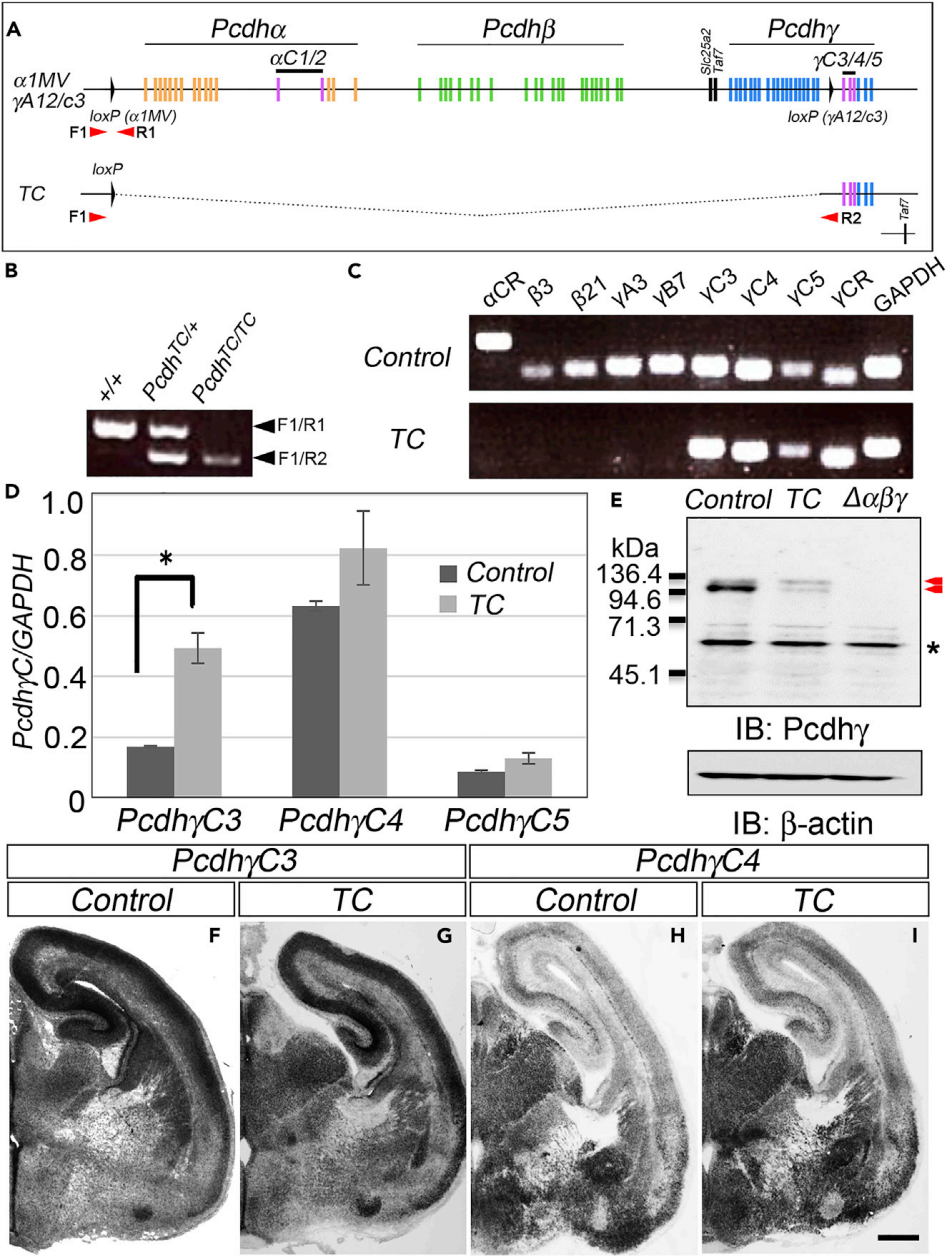
*TC* mutants maintain normal expression of the three constitutive γC-type isoforms (A) Genetic organization of the *TC* mutant allele. The genomic positions of *α1MV loxP* and *γA12/C3 loxP* insertion sites (upper diagram, see also Figure S1). *TC* mutants were generated by Cre-induced miotic recombination at *α1MV* and *γA12/C3 loxP* sites (lower diagram). Arrows indicate primer positions used for genotyping. (B) PCR genotyping to distinguish between wild-type (+/+) and *TC* mutant alleles. (C) RT-PCR analysis showing the expression of *γC3*, *γC4*, and *γC5* but not the other isoforms in the deleted clusters. αCR and γCR indicates constant region of *Pcdhα* and *γ*, respectively. (D) Quantitative real-time PCR showing the comparable expression of the three *γC*-type isoforms to control mice (*+/+:TG*^*taf7*^), although an increase in *γC3* expression was noted. *N* = 3 animals per genotype. Error bars represent SEM *∗*p < 0.05 by *t*-test. (E) Western blot detection of Pcdhγ isoforms in E18.5 brain lysates from *TC* mutants by the antibody against the constant region of Pcdhγ (anti-γCR, indicated by red arrowheads). cPcdh-null mutants (*Δαβγ*) did not express Pcdhγ, whereas *TC* mutants exhibited a large reduction but still detectable expression from the remaining *γC3*, *γC4*, and *γC5* loci. (F-I) *In situ* hybridization with a *γC3* (F, G) or *γC4* (H, I) cRNA probe on coronal sections of E18.5 control (F, H) or *TC* mutant (G, I) brain. Scale bar: 500 μm.

A genetically modified *TC* (triple γC-type) mutant mouse, which lacked 55 cPcdh isoforms from *α1* to *γA12* but retained only the three C-type isoforms of *Pcdhγ*, was generated (Figure 1). Briefly, we introduced *loxP* sites upstream of *α1* (*loxP-α1MV*)^22^ and downstream of *γA12* (Figure 1A; *loxP-γA12/C3*) by homologous recombination of targeting vectors (Figure S1). A deletion allele lacking the 55 isoforms from *α1* to *γA12* was generated by Cre-induced meiotic recombination by crossing with mice carrying the *Sycp-Cre* transgene (Figures 1A and 1B). This deletion protocol also deleted the essential gene *Taf7* located between the *Pcdhβ*- and *γ*-clusters, the loss of which is known to be early embryonic lethal.^36^ To restore the additionally deleted *Taf7* gene, *TC* mutant mice were crossed with transgenic mice that harbor the *Taf7* transgene (*TG*^*taf7*^).^22^ All mice used in this study, including the control (*+/+:TG*^*taf7*^) and mutants (*TC*, *Δγ,* and *Δαβγ*), carry the *Taf7* transgene.

We initially examined the expression of the retained triple γC-type isoforms in *TC* mutants by reverse transcription-polymerase chain reaction (RT-PCR) (Figure 1C). We confirmed the expression of the *γC* isoforms and that the other isoforms in the deleted region (*αCR*, *β3, β21*, *γA3, γB7*) were not expressed in *TC* mutants (Figure 1C). Since the deletion of the genomic region 5′ upstream of the *γC*-type isoforms or the absence of the other 55 isoforms may affect and change the expression of the three *γC*-type isoforms, we conducted quantitative real-time PCR. The expression of the *γC*-type isoforms in *TC* mutants was comparable with that in control (*+/+:TG*^*taf7*^); no significant change in *γC4* and *γC5* was observed, whereas higher expression was noted in *γC3* (Student’s *t* test, p < 0.05; Figure 1D). We also examined the spatial expression of *γC3* and *γC4* mRNA by *in situ* hybridization (ISH). As shown in Figures 1F-1I, spatial expression patterns of *γC3* and *γC4* in control mice were retained and did not change in *TC* mutants. Both isoforms were widely expressed in the brain with more prominent expression of *γC3* in the cerebral cortex and higher expression of *γC4* in the thalamic region (Figures 1F-1I). The expression of the Pcdhγ protein was probed with the antibody against the constant region of Pcdhγ. The antibody detected all Pcdhγ protein isoforms in the control lysate, which were completely absent in the *Δαβγ* mutant. The antibody detected Pcdhγ expression in the *TC* mutant (Figure 1E), confirming that the three γC-type isoforms were translated into proteins. The amount of Pcdhγ protein in the *TC* mutant was low, which is consistent with the result of quantitative real-time PCR. The large protein reduction was due to the deleted γA- and γB-type isoforms and the remaining signal was due to the maintained expression of triple γC-type protein products (Figure 1E).

**Figure 2:**
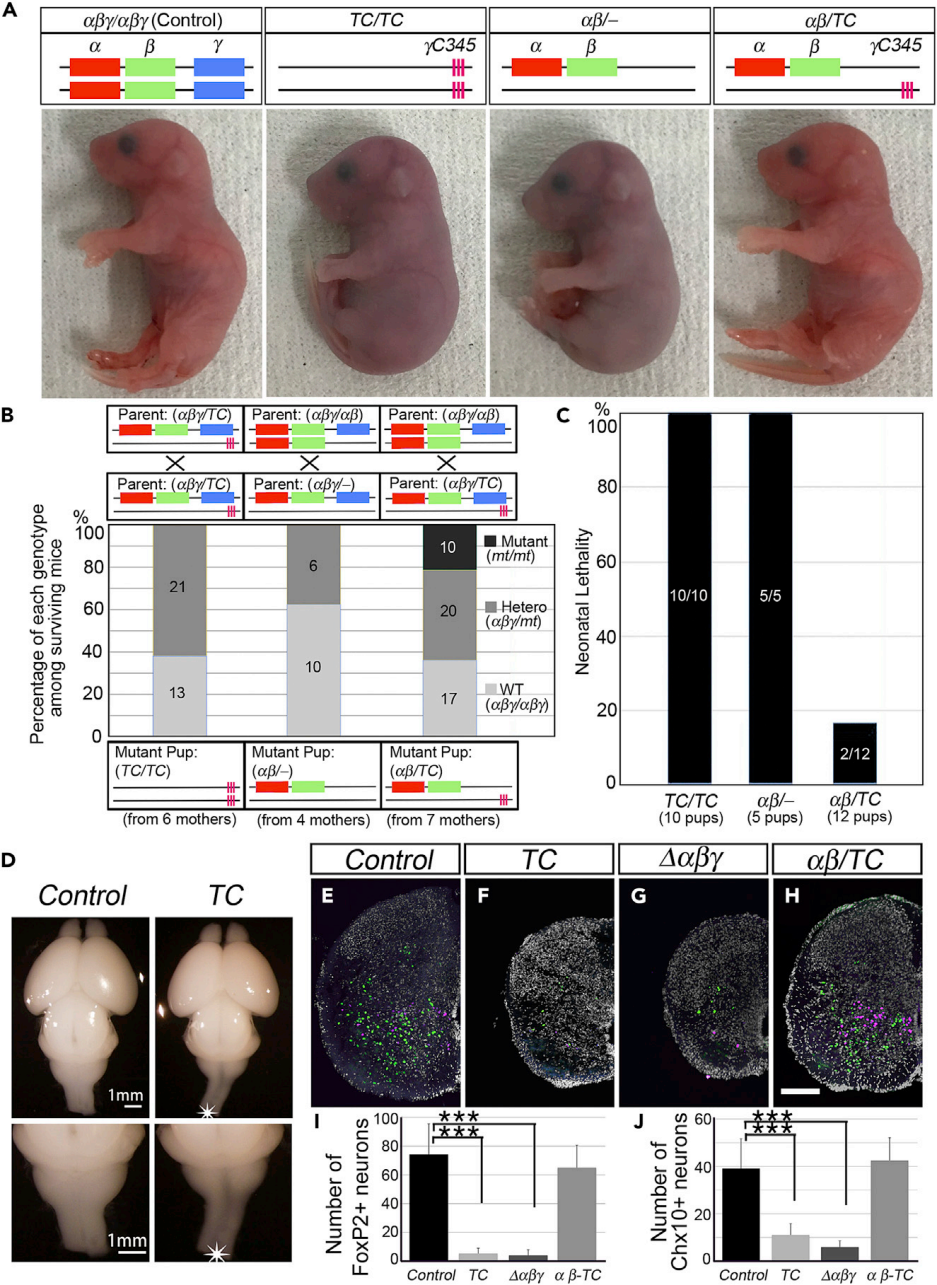
*TC* mutants died after birth, and neuronal loss was observed in the spinal cord (A) Gross phenotypes of P0/E19.5 neonatal mice. Mouse genotypes are indicated at the top to represent the retained *Pcdh* cluster or isoform name for each allele. *TC* mutants (*TC/TC*) exhibited a hunched posture and umbilical hernia in most cases and died immediately after birth. The *αβ*/- mice (lacking γ-cluster) also died after birth, but the *αβ/TC* mice survived. See also Video S1. Response of a neonatal control mouse (αβγ/αβγ) to tail pinch, related to Figure 2, Video S2. Response of a neonatal TC mutant mouse (TC/TC) to tail pinch, related to Figure 2, Video S3. Response of a neonatal mutant mouse harboring only a single allele of α- and β-clusters (αβ/-) to tail pinch, related to Figure 2, Video S4. Response of a neonatal mutant mouse harboring a single allele of α- and β-clusters and TC allele (αβ/TC) to tail pinch, related to Figure 2. (B) Percentage of each genotype among the mice surviving 1 h after natural birth or Caesarean delivery. Parent genotypes are indicated at the top, and the resulting mutant genotypes (both mutant alleles) are at the bottom. Parent combinations to generate each mutant were as follows: for *TC* mutant, *αβγ/TC* × *αβγ/TC*; for *αβ*/- mutant, *αβγ*/*αβ* × *αβγ*/-; and for *αβ/TC* mutant, *αβγ*/*αβ* × *αβγ/TC*. Graph legend (mt) represents either mutant allele from mated parents. Numbers in the graph indicate the number of mice examined. (C) Percentage of neonatal lethality in mice with indicated genotype. (D) Whole brains of E18.5 *TC* mutants. Note the thinner spinal cord of *TC* mutants compared with control mice (asterisks). (E-H) Transverse sections of E18.5 spinal cords immunostained for FoxP2 (green) and Chx10 (magenta). Scale bar: 100 μm. (I, J) Neuron counts for FoxP2^+^- (I) and Chx10^+^- (J) neurons in the ventral spinal cord. *N* = 7 animals for control (*+/+:TG*^*taf7*^), *N* = 4 for *TC* (*TC/TC*) and for *Δαβγ*, and *N* = 5 for *αβ*/*TC* mutant (five sections per animal). Data are represented as mean ± SD *∗∗∗*p < 0.001 by one-way analysis of variance and Tukey’s *post-hoc* test.

Subsequently, we examined neonatal lethality, which was the salient common phenotype among all the mutant mice that lacked the critical γC4 isoform, such as the *Δγ*, *Δβγ*, *Δαβγ*, *TCKO* mutants, and *Δγ*C4 mutant.^22^^,^^33^^,^^35^^,^^37^ *TC* mutant mice were born alive; however, they exhibited acromphalus, a hunched posture, shallow breathing, slight movement, and no response to any touch of physical stimuli. Due to severely impaired breathing and blood circulation, the mutants died immediately after birth despite the confirmed expression of the three γC-type isoforms (Figures 2A-2C; *TC/TC*, Videos S1, and S2). Their phenotype resembled that of *Δαβγ* and was more severe than that of *Δγ* mutants, which exhibited a repetitive limb tremor and died within 12 h after birth (Video S1 in Hasegawa et al.^22^). To confirm that the three γC-type isoforms in *TC* mutants were functional, we examined whether the *TC* allele could rescue the neonatal lethality of mutant mice that lacked the three γC-type isoforms. The mutant *αβ/-* mice lacking *Pcdhγ* (which was generated by crossing *αβγ/αβ* mice with *αβγ/-* mice and retained only a single allele of *Pcdhαβ*) died after birth (Figures 2A-2C; *αβ/-*). The behavioral defect of *αβ/-* mice was more severe than that of *Δ*γ mice (*αβ/αβ*). The mice exhibited little movement and little response to physical stimuli and died immediately after birth (Video S3). The introduction of a single *TC* allele into *αβ/-* mutant mice, such that they harbored a single allele of *Pcdhαβ* and a single allele of *TC* (*αβ/TC*), rescued neonatal lethality (Figures 2A-2C; *αβ/TC*). The *αβ/TC* mice were born alive and behaved normally, providing proof of *TC* allele functionality (Video S4). The *αβ/TC* mice survived beyond 7 months and were fertile, although their body weights were less than their littermates (at 7 weeks, the body weight of three *αβ/TC* males was 18.5 g ± 0.5 g, whereas the average weight of the other nine males was 23.1 g ± 1.0 g; the average body weight of four *αβ/TC* females was 16.8 g ± 0.8 g, whereas the average weight of the other eleven females was 19.2 g ± 1.4 g). The above results clearly showed that the deletion of 55 isoforms was neonatal lethal despite maintaining the functional *TC* allele and also showed that the three γC-type isoforms require the other isoforms in the *Pcdhα* and *Pcdhβ* clusters for the survival of the mice.

Video S1. Response of a neonatal control mouse (αβγ/αβγ) to tail pinch, related to Figure 2

Video S2. Response of a neonatal TC mutant mouse (TC/TC) to tail pinch, related to Figure 2

### Massive apoptotic cell death in the brainstem reticular formation in *TC* mutants

The lack of the three γC-type isoforms (*TCKO*) was neonatal lethal but also caused massive apoptotic cell death in the spinal cord^33^^,^^37^^,^^38^ and brainstem.^22^ We, therefore, examined whether apoptotic cell death also occurs in *TC* mutants.

The spinal cords of *TC* mutants were thinner, suggesting a reduction in neuronal numbers (Figures 2D-2F). Apoptosis was quantified by counting the remaining neurons of two representative neuronal types, namely, V1 inhibitory interneurons (FoxP2-expressing subsets) and V2a excitatory interneurons (Chx10-expressing subsets). The number of surviving FoxP2(+) interneurons and Chx10(+) interneurons in *TC* mutants was approximately 7.1 and 28.0% that of control mice, respectively, which was comparable to the reduction observed in *Δαβγ* mutant mice^22^ (Figures 2I and 2J). Mutant mice carrying a combination of one *TC*-allele and one *αβ*-allele (*αβ/TC* mice) exhibited a normal spinal cord diameter. There was no statistically significant difference in the number of the FoxP2(+) and Chx10(+) interneurons between the control and *αβ/TC* mice (Figures 2H-2J), indicating that *γC3*, *γC4*, and *γC5* in the *TC*-allele are functional for neuronal survival.

**Figure 3:**
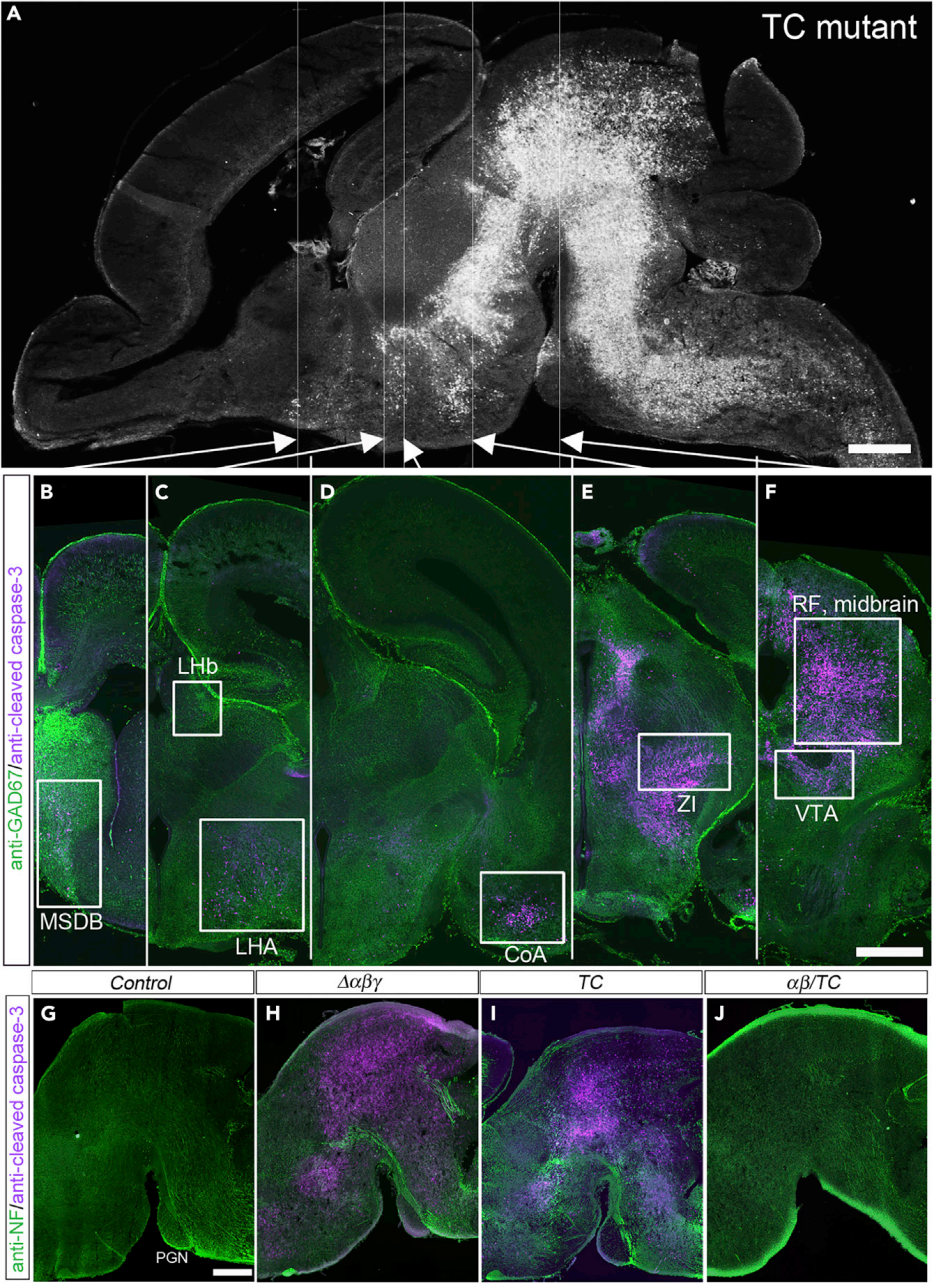
Massive apoptosis occurred in the brainstem of *TC* mutants (A) A sagittal section of the whole brain of E18.5 *TC* mutant, immunostained for cleaved-caspase-3 (CC3) (white). Massive cell death occurred in the brainstem. CC3 was expressed not only in the soma but also in degenerating dendrites and axons. Therefore, the degenerating fiber tracts were also stained. (B-F) Coronal sections of the brain of E18.5 *TC* mutant immunostained for CC3 (magenta) and GAD67 (green). Sections were arranged in rostro-caudal order from left to right. Rectangle indicates the area exhibiting apoptosis. The rostro-caudal position of each coronal section is indicated by arrows overlaid in the sagittal section in Figure A. (G-J) Sagittal sections of E18.5 midbrains immunostained for CC3 (magenta) and neurofilament (green). When *TC* alleles were complemented with a single allele of *Pcdhαβ* (*αβ/TC* mutant), apoptotic neuronal death was completely suppressed (J). MSDB, medial septum-diagonal band; LHb, lateral habenular nucleus; LHA, lateral hypothalamic area; CoA, cortical amygdala; ZI, zona incerta; RF, reticular formation; VTA, ventral tegmental area. Scale bars: 500 μm in (A), 500 μm in (B-F), and 250 μm in (G-J).

**Figure 4:**
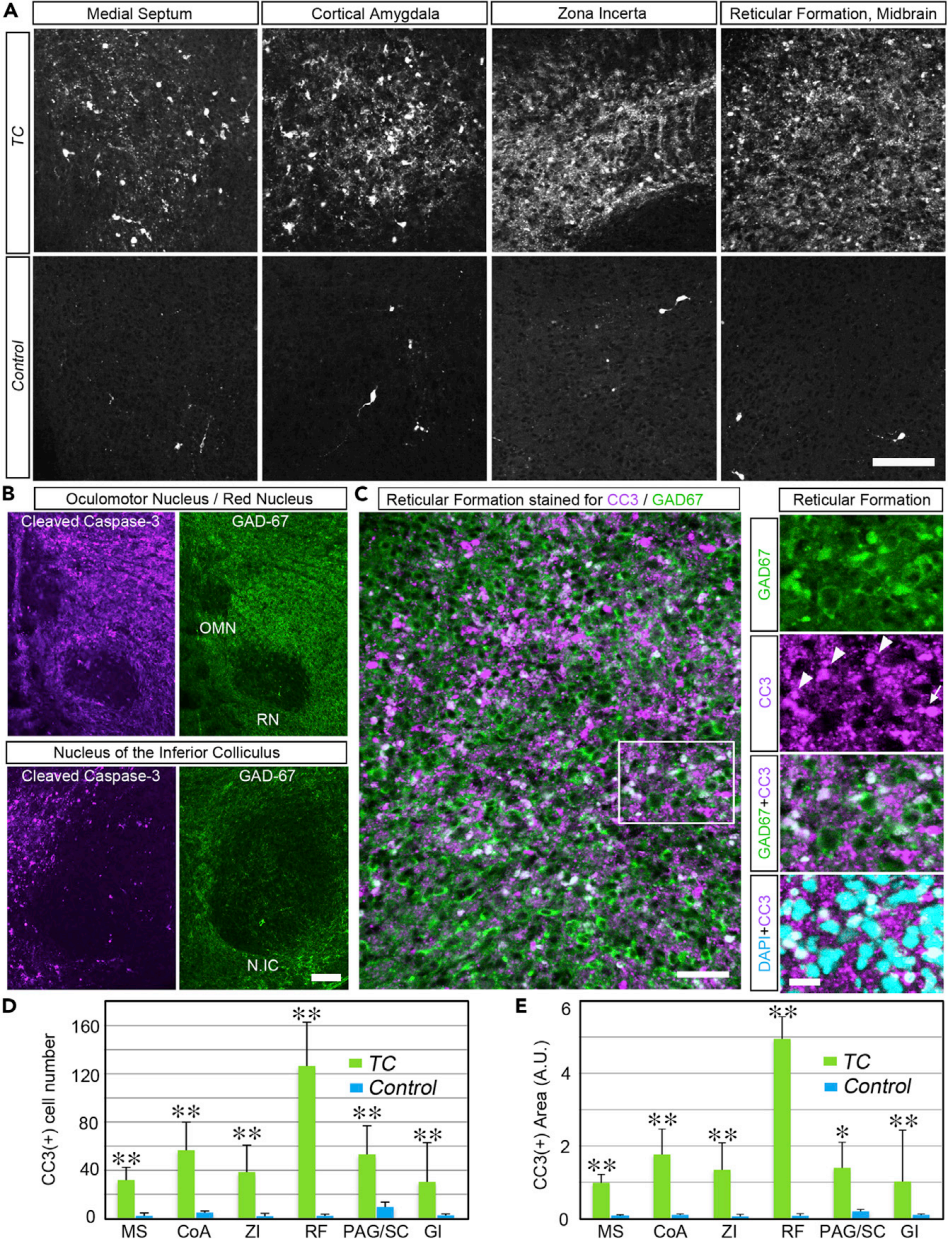
Enhanced rate of apoptosis in specific nuclei in *TC* mutants (A) Higher magnification views of brain areas exhibiting apoptotic cell death in the coronal section of E18.5 *TC* mutant and control mice (*+/+:TG*^*taf7*^). Sections were immunostained for CC3. (B) Spatial correlation of apoptosis and GAD67 distribution. Paired image of the same visual field immunostained for CC3 (magenta, left) and GAD67 (green, right). Brain areas devoid of massive apoptotic cell death in *TC* mutants stained less for GAD67. (C) (Left) Reticular formation of *TC* mutants double immunostained for CC3 (magenta) and GAD67 (green). (Right) Higher magnification views of the section indicated by the rectangle in the left image, triple stained for GAD67 (green), CC3 (magenta), and DAPI (cerulean). The arrowhead and arrow indicate GAD67(+) and GAD67(−) cells, respectively. DAPI staining was performed to show the location of the cell body. (D) Quantification of apoptotic cell counts in the fixed region of interest (ROI) for each brain area. CC3 signal with cell somatic diameter that matched with DAPI staining was counted as a cell. Data are represented as mean ± SD. *N* = 5 animals per genotype. *∗∗*p < 0.01 by Mann-Whitney *U*-test. (E) Quantification of CC3-stained area (including soma and degenerating neuronal processes) in the fixed region of interest (ROI) for each brain area. The total area of pixels with the above-threshold intensity was measured. Data are represented as mean ± SD. *N* = 5 animals per genotype. *∗∗*p < 0.01, *∗*p < 0.05 by Mann-Whitney *U*-test. Scale bars: 100 μm in (A), 100 μm in (B), 50 μm in (C, left), and 20 μm in (C, right).

Next, we examined apoptotic cell death in the brain of an E18.5 *TC* mutant embryo. Figure 3A shows a sagittal section of the whole brain of a *TC* mutant embryo stained for cleaved-caspase-3 (CC3), a marker of apoptotic cells. Massive cell death was observed in the brainstem (midbrain, pons, medulla oblongata) (Figure 3A). Apoptotic cells were also observed in several specific nuclei in the forebrain (Figures 3B-3E, higher magnification in Figure 4A; Table 1). Examination of coronal sections revealed apoptotic cells in the medial septum-diagonal band (MSDB) (Figures 3B and 4A), lateral habenular nucleus (LHb) (Figure 3C), lateral hypothalamic area (LHA) (Figure 3C), ventral edge of the amygdala (future cortical amygdala, or cortex-amygdala transition zone, hereafter designated as cortical amygdala or CoA) (Figures 3D and 4A), zona incerta (ZI) (Figures 3E and 4A), midbrain reticular formation (Figures 3F and 4A), ventral tegmental area (VTA) (Figure 3F), the area dorsal to the aqueduct including periaqueductal gray (PAG) and superior colliculus, and in the gigantocellular nucleus (Table 1). Co-immunostaining of CC3 and the inhibitory neuronal marker GAD67 showed a correlation between the apoptotic cell death area and GAD67-enriched area. Apoptotic cells were normally observed in GAD67-enriched regions, such as the septum and ZI (Figures 3B and 3E). Conversely, brainstem nuclei with weak GAD67 expression, such as the oculomotor nucleus, red nucleus, and the nucleus of the inferior colliculus, appeared to be devoid of (or had fewer) apoptotic cells (Figure 4B). A subfraction of CC3-positive cells was also GAD67-positive, suggesting that inhibitory neurons were undergoing an apoptotic process (Figure 4C). Quantitative analysis of apoptotic cell numbers or apoptotic cell area (including the area occupied by degenerating neuronal processes) showed that apoptosis also occurred with low frequency in control mice, whereas in *TC* mutant mice, the frequency increased by more than 10 times (Figures 4A, 4D and 4E). Among the brain regions, the midbrain reticular formation was the most severely affected. Analysis of apoptotic cell distribution suggests that apoptosis occurred in the brainstem reticular formation-centered interconnected neural networks with enriched inhibitory connections.

**Table 1:** List of brain areas where massive or little apoptosis was observed in *TC* mutants

Area/Nucleus	Apoptosis
Medial septum	+++
Nucleus of the vertical limb of the diagonal band	+++
Lateral hypothalamic area	++++
Lateral habenular nucleus	++
Cortical amygdala	++++
Zona incerta (caudal area)	++++
Precommissural nucleus	++
Periaqueductal gray	++++
Ventral tegmental area (A10)	+++
Substantia nigra (A9) reticular part	++
Reticular formation, midbrain	+++++
Oculomotar nucleus	void
Red nucleus	void
Superior colliculus	+++
Reticular formation, pons	+++
Nucleus of the inferior colliculus	void
Gigantocellular nucleus	+++
Raphe magnus nucleus (B3)	++
Inferior olive	void

“Void” indicates that apoptosis occurred significantly less in these regions compared with the neighboring regions where massive apoptosis was observed.

### Spatially overlapping expression of cPcdh stochastic and *γ*C4 isoforms

**Figure 5:**
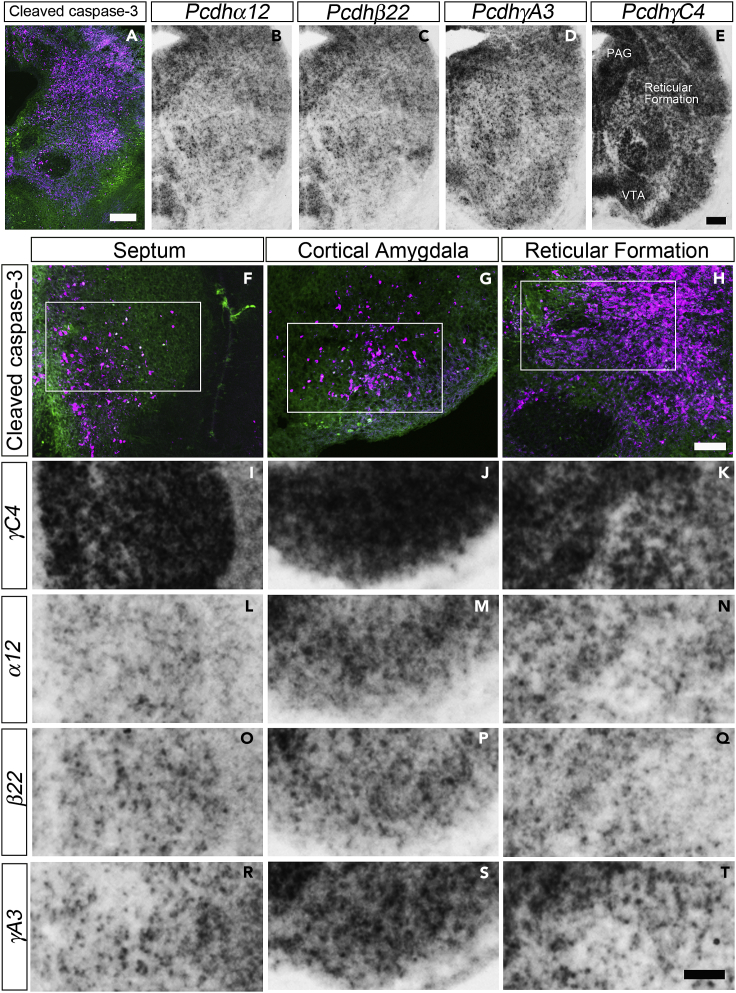
Nuclei susceptible to apoptosis in *TC* mutants exhibited the combinatorial expression of dominant *γC4* and stochastic isoforms (A) A coronal section of the midbrain of an E18.5 *TC* mutant at the level of the red nucleus double-stained for CC3 (magenta) and GAD67 (green). Massive apoptosis occurred at the reticular formation. (B-E) *In situ* hybridization (ISH) with *α12* (B), *β22* (C), *γA3* (D), and *γC4* (E) antisense probes in the coronal sections of the midbrain of a control mouse (*+/+:TG*^*taf7*^). Sections in B-E were all neighboring sections from the same brain. (F-T) Magnified view of the three representative brain areas showing massive apoptosis: septum (F, I, L, O, R), cortical amygdala (G, J, M, P, S), and reticular formation in the midbrain (H, K, N, Q, T). (F-H) Double immunostaining for CC3 (magenta) and GAD67 (green). Rectangles are approximate visual fields of images in (I-T). (I-T) ISH of *γC4* (I-K), *α12* (L-N), *β22* (O-Q), and *γA3* (R-T) antisense probes in the coronal sections of E18.5 control brain corresponding to the rectangular area in (F-H). ISH signal clearly showed the dominant expression of *γC4* in the vast majority of cells and the sparse expression of *α12*, *β22*, and *γA3* stochastic isoforms. Scale bars: 200 μm in (A), 200 μm in (B-E), 100 μm in (F-H), and 100 μm in (I-T).

The viability of *αβ/TC* mice, in contrast to the neonatal death of *TC* mutants (Figures 2A-2C), suggested that the critical *γ*C4 isoform needs to work in concert with the stochastic isoforms to exert its function. To elucidate the relationships of the spatial expression pattern of the stochastic isoforms and the *γ*C4 isoform, we conducted ISH of representative stochastic isoforms (*α12*, *β22*, *γA3*) and the *γC4* isoform (Figure 5). *γC4* isoforms were highly expressed in the wide areas in the midbrain including PAG, midbrain reticular formation, and VTA (Figure 5E), where massive apoptosis was observed in the *TC* mutant (Figure 5A). Stochastic (*α12*, *β22*, *γA3*) isoforms were also expressed in similar areas in the midbrain of control mice, but the expression level of stochastic isoforms was generally very low and sparse (Figures 5B-5D). Figures 5F-5T show representative examples of three brain areas where apoptosis was observed in *TC* mutants. As clearly shown in the higher magnification view of the septum (Figures 5F, 5I, 5L, 5O, and 5R), cortical amygdala (Figures 5G, 5J, 5M, 5P, and 5S), and the reticular formation in the midbrain (Figures 5H, 5K, 5N, 5Q, and 5T), the *γC4* isoform was expressed in the vast majority of cells in these regions (Figures 5I-5K), whereas the *α12*, *β22*, and *γA3* isoforms were stochastically and sparsely expressed (Figures 5L-5T). This expression pattern was also observed in other brain areas, such as the LHb and the VTA (data not shown). These results clearly show that the corresponding control brain regions where massive apoptosis occurred in *TC* mutants not only expressed the essential *γ*C4 but also invariably expressed the stochastic isoforms simultaneously.

**Figure 6:**
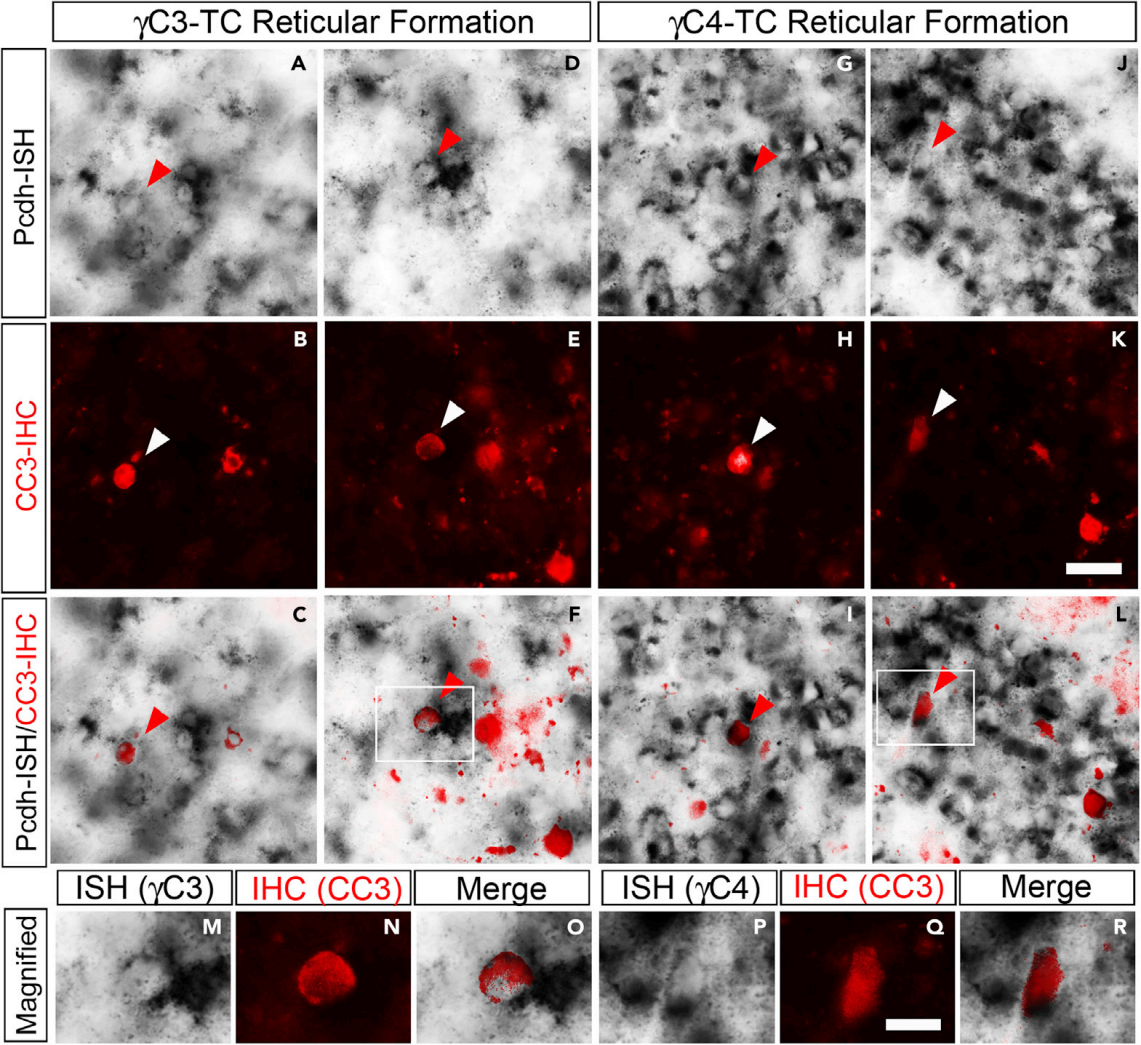
Expression of *γC3* or *γC4* did not protect cells from apoptosis in *TC* mutants (A-L) Dual staining for *in situ* hybridization (ISH) for γC-type isoforms (gray scale) (A, D, G, J) and immunostaining for CC3 (red) (B, E, H, K) of the brain sections of the reticular formation of an E18.5 TC mutant. (C, F, I, L) Merged images of CC3 signals overlaid on ISH signals. Both γC3-expressing cells (A-F, arrowheads) and γC4-expressing cells (G-L, arrowheads) were undergoing apoptosis. (M-R) Magnified view of γC3-expressing cells (M−O) in the rectangular area in (F), and γC4-expressing cells (P-R) in the rectangular area in (L), expressing the apoptotic marker CC3. Scale bars: 20 μm in (A-L) and 10 μm in (M-R).

Finally, we examined whether apoptotic cells expressed the *γC*-type isoforms. For this purpose, we conducted dual immunohistochemistry (IHC) for CC3 and ISH for *γC3* or *γC4* mRNAs. The spatial expression pattern of the *γC*-type isoforms in the *TC* mutant did not change and was the same as that in control mice (Figures 1F-1I). Figure 6 shows a higher magnification view of the ISH signal in the midbrain reticular formation for *γC3* (Figures 6A and 6D) and *γC4* (Figures 6G and 6J). The density of *γC3*-expressing cells was a little low compared with *γC4*, but quite a few cells expressed *γC3* (Figures 6A and 6D). As the cells positive for CC3 were undergoing an apoptotic process, the mRNAs in these cells were potentially in the process of degradation. However, we found cells expressing both *γC3* and CC3 (Figures 6C and 6F, higher magnification in 6M−6O) and cells expressing both *γC4* and CC3 (Figures 6I and 6L, higher magnification in 6P-6R). This result suggests that the expression of either *γC3* or *γC4* did not ensure cell viability in the *TC* mutant.

## Discussion

We examined the phenotype of *TC* mutant mice lacking 55 cPcdh isoforms except the essential three *γC*-type isoforms. The mice died immediately after birth and exhibited massive neuronal death despite the presence of the functional *TC* allele. This finding suggests that the three *γC*-type isoforms were not sufficient to rescue the neuronal defects of clustered Pcdh-null mice (*Δαβγ*), and other cPcdh isoforms are also required for the survival of neurons and individual mice.

Apoptosis was also observed in control mice in the same brain regions as *TC*, albeit the frequency of apoptotic cells was very low in control (Figures 4A and 4C). This suggests that apoptotic cell death is a normal developmental process that occurs during neural network wiring. Massive apoptosis occurs in the absence of cPcdh (*Δαβγ* mouse),^22^ suggesting that the default strategy of neural network formation in the brainstem and spinal cord is to exclude inappropriate cells by apoptosis and that cPcdh provides the survival signal. PcdhγC4 has been shown to be essential for the survival signal.^33^^,^^35^ However, in *TC* mutants, the expression of PcdhγC4 did not ensure neuronal survival. At least the following three explanations are possible.

The first possibility is that PcdhγC4 is nonfunctional in *TC* mutants due to the failure in cell surface trafficking, It has been shown that certain cPcdh isoforms including PcdhγC4, the stochastic isoforms in Pcdh*α* (*α*1-12) and Pcdh*α*C1, cannot translocate to the cell surface by themselves (when expressed alone). PcdhγC4 requires another carrier isoform to form a *cis*-dimer to enable its translocation to the cell surface.^11^^,^^12^^,^^17^ *TC* mutants express both *γ*C3 and *γ*C5 isoforms as potential carriers. However, *γ*C5 expression was low at the prenatal stage, and the expression of *γ*C3 was, to our surprise, rather sparse compared to *γ*C4 in the apoptotic brain regions. Therefore, in *TC* mutants, the remaining *γ*C3 and *γ*C5 may not be enough for *γ*C4 cell surface expression. This must be addressed in the future.

A second possibility is that the deleted isoforms in *TC* (*Pcdhα1* to *PcdhγA12*) are also critical for the survival of neurons and individual mice. Although the *γ*C4 appeared as the only critical isoform, requirement of other isoforms has also been reported. The severity of apoptosis in the *Δγ* mutant was augmented by the additional deletion of *Pcdhα* and/or *Pcdhβ*, suggesting the contribution of *α*- and *β*-isoforms.^22^^,^^39^ Ing-Esteves et al., reported dose- (allele number) dependent effects of *Pcdhα* and *Pcdhγ* cluster deletions on retinal survival.^39^ We also observed the allele number-dependent effects of *Pcdhα* and *Pcdhβ* clusters on neonatal survival; the *Δγ* mutant (*αβ*/*αβ*) exhibited repetitive limb tremor and died within 12 h, while the *αβ*/- mutant exhibited little movement and died immediately after birth. The requirement of *γ*C4 might be attributable to it being the most dominantly expressed isoform in the apoptotic brain areas. The simultaneous loss of a bunch of other stochastic isoforms with low expression may also cause the lowering of the survival signal below the required threshold.

A third possibility is that the *cis*-dimer of PcdhγC4 and another isoform acts as the functional unit and exerts its antiapoptotic signal. A disadvantageous feature of *γ*C4 is that it alone cannot be transported to the cell surface and requires another isoform (forming a *cis*-heterodimer) for its cell surface delivery. Therefore, the generation of the survival signal of γC4 is linked to the diversity of cell-surface recognition ability. Our expression study at E18 indicated that the essential γC4 isoform is always expressed with other stochastic isoforms, which is consistent with the above idea. The defect observed in *TC* mutants supports that the γC4 requires other isoforms (included in deleted 55 isoforms). Whether there is a specific carrier isoform, or in fact 55 isoform variety is required, awaits further study.

Neuronal types whose survival depends on cPcdh have been reported in many brain areas, such as spinal cord interneurons, brainstem neurons, cortical interneurons, retinal neurons.^22^^,^^32^^,^^34^^,^^38^^,^^39^^,^^40^ Here, we mapped the cPcdh-dependent neurons in the forebrain and midbrain at E18. The cell death areas include the MSDB, LHb, LHA, cortex-amygdala transition zone, ZI, VTA, PAG, and midbrain reticular formation, which were directly connected according to the previous studies.^41^^,^^42^^,^^43^^,^^44^^,^^45^ Therefore, apoptotic cell death area appeared as a directly connected single mass of neural network. This suggests that the apoptotic cell death in *TC* mutants was correlated with network organization and may be correlated with its activity.

GABAergic interneurons are a major apoptotic cell type found in the *Δγ* mutant. There exists a correlation between the severity of neuronal loss (reduction volume of the tissue) and abundance of GABAergic neurons (*GAD2* expression).^22^^,^^34^^,^^37^^,^^38^^,^^40^ We also found that the spatial distribution of CC3-positive apoptotic cells in the E18 *TC* mutant closely resembles that described in published ISH data for *GAD1/GAD2* (available on Allen Brain Atlas; https://portal.brain-map.org).^46^ The distribution of CC3-positive cells in *TC* mutants also resembles (although not exactly) the staining pattern of acetylcholine esterase (AChE) in the coronal plane of the E15/16 mouse brain (as reported in the mouse brain atlas by Jacobowitz and Abbott^47^). These correlations suggest that the cell death area is enriched with GABAergic neurons and generally receives cholinergic input.

It has been shown that synchronized rhythmic activity, which is distinct from the activity of a mature circuit, occurs at many sites in the developing nervous system.^48^ This activity has been extensively studied in the spinal cord; a wave of the synchronized rhythmic activity is propagated over the entire network, driven by both GABAergic and cholinergic inputs, and has been considered a general necessary program of neural circuit wiring.^48^^,^^49^^,^^50^^,^^51^^,^^52^^,^^53^ Massive apoptosis in the developing spinal cord of *Pcdh*-deficient mice (such as *TC* and *Δαβγ*) occurred after the cessation of synchronized rhythmic activity.^22^^,^^53^ The close spatial correlation of apoptotic cell distribution in *TC* mutants with that of GABAergic neurons and AChE and the temporal correlation of the onset of apoptosis and the cessation of a synchronized rhythmic activity suggest that embryonic rhythmic activity may be a prerequisite for the cPcdh-mediated survival signal. Determining whether synchronized neuronal activity plays a role in cell surface trafficking of cPcdh, which generates the survival signal, or whether the activity of the network whose wiring is regulated by cPcdh itself governs neuronal survival, requires further study. Taken together, the γC-type isoforms appeared to regulate neuronal survival by cooperating with other cPcdh isoforms, the molecular mechanism of which should be clarified in the future.

### Limitations of the study

In this study, we have shown that the three PcdhγC-type isoforms (γC3, γC4, γC5) were insufficient to prevent neuronal apoptosis and neonatal death of *TC* mutant mice. This result suggests that the critical γC4 isoform requires other Pcdh isoforms to play a role in the survival of neurons and mice. However, the molecular mechanisms are still unclear. The cell surface expression of γC4 isoform, the actual entity of *cis*-dimer/multimer Pcdh complex, and the downstream signaling cascade need to be clarified in the future. The differential role of γC4 and other stochastic isoforms will be elucidated in the future by re-introducing stochastic isoforms to the *TC* allele.

## STAR★Methods

### Key resources table

**Table:** 

REAGENT or RESOURCE	SOURCE	IDENTIFIER
**Antibodies**		

Anti-cleaved-caspase-3	Cell Signaling Technology	#9661; RRID:AB_2341188
Anti-GAD67	Millipore	#MAB5406; RRID:AB_2278725
Anti-Chx10	Santa Cruz	#sc-21690; discontinued
Anti-FoxP2	Sigma-Aldrich	#HPA000382; RRID:AB_1078908
Anti-pan axonal-neurofilament (SMI312)	Covance	#SMI312; discontinued
Guinea pig anti-PcdhγCR antibody	produced by CBSN, Hasegawa et al. (2016)	N/A
Anti-β-actin	Sigma-Aldrich	#A5441; RRID:AB_476744
Anti-digoxigenin-AP Fab fragments antibody	Sigma-Aldrich	#11093274910; RRID:AB_2734716
Goat anti-mouse IgG Alexa Fluor Plus 488	ThermoFisher	#A32723; RRID:AB_2633275
Goat anti-rabbit IgG Alexa Fluor Plus 594	ThermoFisher	#A32740; RRID:AB_2762824
Donkey anti-goat IgG Alexa Fluor 594	ThermoFisher	#A11058; RRID:AB_2534105
Donkey anti-rabbit IgG Alexa Fluor 488	ThermoFisher	#A21206; RRID:AB_2535792

**Chemicals, peptides, and recombinant proteins**		

TRIzol reagent	ThermoFisher	Cat#15596018
DNaseI (RNase-free)	TaKaRa Bio, Inc.	Cat#2270A
Superscript III reverse transcriptase	ThermoFisher	Cat#18080093
SYBR Premix Ex Taq	TaKaRa Bio, Inc.	Cat#RR420A, discontinued
ECL Select	Cytiva	Cat#RPN2235
Diethyl pyrocarbonate	Nacalai Tesque, Inc.	Cat#12311-86
Ribonuclease inhibitor (porcine liver)	TaKaRa Bio, Inc.	Cat#2311A
T7 RNA polymerase	Promega	Cat#PR-P2075
T3 RNA polymerase	Promega	Cat#PR-P2083
DIG RNA labeling mix	Sigma-Aldrich	Cat#11277073910
ProtectRNA RNase inhibitor	Sigma-Aldrich	Cat#R-7397
Formamide, deionized, nuclease and protease tested	Nacalai Tesque, Inc.	Cat#16345-65
4-Nitro blue tetrazolium chloride	Roche	Cat#11383213001
5-Bromo-4-chloro-3-indoyl-phosphate	Roche	Cat#11383221001
Paraformaldehyde	Nacalai Tesque, Inc.	Cat#02890-45
Low melting temperature gelatin	Nippi	Cat#MAX-F
Tissue-Tek O.C.T. compound	Sakura Finetek	N/A
Immunoselect antifading mounting medium	Dianova	Cat#SCR-038447
Proteinase K	Nacalai Tesque, Inc.	Cat#29442-85

**Experimental models: Organisms/strains**		

Mouse: C57BL/6 *Pcdhabg*^TC/+^:TG^*Taf7*^	This study	RIKEN BRC, RBRC09813
Mouse: C57BL/6 *Pcdhabg*^del/+^:TG^*Taf7*^	Hasegawa et al. (2016)	RIKEN BRC, RBRC04820
Mouse: C57BL/6 *Pcdhg*^del/+^:TG^*Taf7*^	Hasegawa et al. (2016)	RIKEN BRC, RBRC04821
Mouse: C57BL/6 TG^*Taf7*^	Hasegawa et al. (2016)	RIKEN BRC, RBRC04822

**Oligonucleotides**		

For Southern blot probe A, B sequence, see Table S1	This study	N/A
For genotyping *Pcdhabg*^TC^ allele, see Table S1	This study	N/A
For genotyping TG^*Taf7*^ allele, see Table S1	This study	N/A
For RT-PCR/real-time RT-PCR, see Table S1	This study	N/A
For amplifying *in situ* hybridization RNA probes, see Table S1	This study	N/A

**Software and algorithms**		

ImageJ ver1.53	NIH	N/A
PRISM 7.05	GraphPad	N/A

**Other**		

ImageQuant LAS-4000	Cytiva	LAS-4000
ABI 7900HT Fast Real-Time PCR System	Applied Biosystems	7900HT
Leica CM3050 cryostat	Leica	CM3050
DS-Qi1Mc digital camera	Nikon	DS-Qi1Mc
BIOREVO BZ-9000 All-in-one Fluorescence Microscope	Keyence Corp.	BZ-9000
Dragonfly	Andor, Oxford Instruments, Belfast, Northern Ireland	https://andor.oxinst.com/products/dragonfly-confocal-microscope-system

### Resource availability

#### Lead contact

Further information and requests for resources and reagents should be directed to and will be fulfilled by the lead contact, Takeshi Yagi (yagi@fbs.osaka-u.ac.jp).

#### Material availability

*TC* mutant mouse lines generated in this study have been deposited to RIKEN BRC (https://web.brc.riken.jp/en/)^54^ (strain: B6; TT2-Pcdh<dla1-gA12>/B6-Tg(Taf7): RBRC09813).

### Experimental model and subject details

#### Animals

All animal procedures were performed according to the Guide for the Care and Use of Laboratory Animals of the Science Council of Japan and were approved by the Animal Experiment Committee of Osaka University and the Institutional Animal Care and Use Committee of RIKEN Kobe Branch. Adult (beyond 2 months old) male and female *Pcdh*^TC/+^:TG^*Taf7*^, *Pcdhabg*^del/+^:TG^*Taf7*^ (Hasegawa et al.^22^), *Pcdhg*^del/+^:TG^*Taf7*^ (Hasegawa et al.^22^), and *Pcdh*^+/+^:TG^*Taf*^ (Hasegawa et al.^22^) mice in a C57BL/6 background were used and maintained in the animal facility of Osaka University. Mice were housed in groups under a 12 h:12 h light:dark cycle. Control mice (*+/+:TG*^*taf7*^) were the littermates of heterozygous breeding for each genotype. All mouse strains used in this study were deposited in RIKEN BRC, Japan.

### Method details

#### Generation of *TC* mutant mice

We introduced *loxP* sites upstream of *α1* (*loxP-α1MV*)^22^ and downstream of *γA12* (*loxP-γA12/C3*; Figures 1A and S1A). Recombinant embryonic stem (ES) cell clones carrying the mutant allele were screened using Southern hybridization (Figure S1, Table S1). The mouse carrying the *loxP-γA12/C3* allele was generated and is maintained at RIKEN BRC (PcdhγA12/C3 (α1MV ES): Accession No. CDB1149K: https://large.riken.jp/distribution/mutant-list.html). A deletion allele lacking 55 isoforms from *α1* to *γA12* (*TC*-allele) was generated by Cre-induced meiotic recombination by crossing with mice carrying *Sycp-Cre* transgene. The initially produced mutants contained an additional deletion of the *Taf7* gene located between the *Pcdhβ* and *Pcdhγ* clusters, the deletion of which is early embryonic lethal.^36^ To rescue the *Taf7* gene, the mutants were crossed with a *TG*^*taf7*^ mouse line containing *Taf7* transgene.^22^ Genotyping of the *TC* allele was performed with primers listed in Table S1: α1-232F and Pcdhα1R1 primers for wild-type allele (721 bp product), and α1-232F and gA12C3intron4846R primers for *TC* allele (553 bp product). *Δγ* and *Δαβγ* mutant mice, both rescued with *Taf7* transgene, were generated, and described in detail in our previous study.^22^ We performed all experiments using *TC* mutant, *Δγ*, *Δαβγ*, and control (+/+:*TG*^*taf7*^) mice.^22^

#### RT-PCR, real-time qRT-PCR, and immunoblot analysis

The primer sequences used for RT-PCR and real-time qRT-PCR are listed in Table S1. Total RNA was extracted using TRIzol Reagent (Invitrogen), and cDNA was synthesized with the Superscript III reverse transcriptase (Invitrogen). The PCR reactions were performed in GC buffer I (TaKaRa, Japan). Quantitative RT-PCR analysis was conducted with SYBR Premix Ex Taq (TaKaRa Bio, Inc., Japan) using ABI 7900HT (Applied Biosystems). Immunoblot analysis was performed as follows. Mouse brains were homogenized in 0.32 M sucrose containing 1 mM EDTA and 1 mM PMSF. The homogenate was spun at 800 × g for 10 min, and the collected supernatant was spun at 20,000 × g for 30 min to obtain the pellet fraction. The pellet fraction was lysed with SDS sampling buffer (60 mM Tris-HCl, pH 6.7, 2% SDS, 2% 2-mercaptoethanol, and 5% glycerol), and the proteins were separated by 7.5% SDS-PAGE. After the proteins were blotted onto nitrocellulose membranes, the membranes were reacted with the following antibodies: guinea pig anti-PcdhγCR antibody (produced by CBSN) and anti-β-actin (Sigma).

#### Neonatal lethality assay

The survival or lethality of P0/E19.5 mice was judged as follows. The survival of P0 pups within 1 h after natural birth was judged by breathing, blood circulation, body color, behavior, and response to tail pinch. As the *TC* mutants died immediately after birth, some mothers abandoned efforts to nurse the other healthy littermates. To exclude the effect of negligence by the mother, we also examined the survival or lethality of E19.5 mice delivered by Cesarean section, similarly judged by breathing, blood circulation, body color, behavior, and response to tail pinch, at 1 hour after resuscitation. Responses to tail pinch of all pups were video recorded.

#### IHC

Embryonic day 16-18 mouse embryos were transcardially perfused with phosphate-buffered saline (PBS) followed by 4% paraformaldehyde (PFA) in PBS. After decapitation and removal of the dorsal cranium to expose the brain, the heads were post-fixed overnight at 4 °C. The brains were then removed, and after cryoprotection in 30% sucrose, they were embedded in O.C.T. compound (Sakura Finetek Co., Ltd., Tokyo, Japan) and quickly frozen in isopentane cooled with liquid nitrogen. Cryosections of 20-μm thickness were cut on a cryostat (Leica CM3050, Germany). IHC was performed with the following antibodies: anti-CC3 (Cell Signaling Technology); anti-GAD67 (Millipore); anti-Chx10 (Santa Cruz); anti-FoxP2 (Sigma), and anti-pan axonal-neurofilament (SMI312, Covance). Secondary antibodies conjugated with Alexa Fluor 488 or 594 were obtained from Molecular Probes.

#### ISH, dual ISH-IHC staining

For ISH, fresh-frozen specimens were used. Briefly, whole brains of E18.5 embryos were dissected out, embedded in 1:2 mixture of 5% fish gelatin in PBS and O.C.T. compound, and immediately frozen in isopentane cooled with liquid nitrogen. After cutting cryosections of 30-μm thickness, sections were post-fixed with 4% PFA for 10 min, acetylated, and hybridized with digoxigenin (DIG)-labeled antisense probes (1-1.5 μg/mL) to each *cPcdh* isoform mRNA at 72 °C overnight. The probe signals were detected using alkaline phosphatase-conjugated anti-DIG antibodies with nitroblue tetrazolium and 5-bromo-4-chloro-3-indolylphosphate as a chromogenic substrate. For the dual staining of ISH and IHC, perfusion-fixed brains with 4% PFA were used. Cryosections of 30-μm thickness were similarly processed and hybridized with DIG-labeled antisense probes. After washing out the probes, sections were incubated with anti-CC3 antibodies and alkaline phosphatase-conjugated anti-DIG antibodies. The alkaline phosphatase color reaction was conducted, followed by the reaction of the Alexa Fluor 488-conjugated secondary antibody to detect the CC3 antibody.

#### Imaging and data analysis

Bright field images were captured using the BX51 microscope (Olympus) equipped with DS-Qi1Mc digital camera (Nikon). Fluorescent images were captured using the BX51 microscope with DS-Qi1Mc, using the BZ9000 microscope (Keyence Corp., Japan), or using the Dragonfly confocal laser microscope (Oxford Instruments, UK). Quantification of FoxP2- or Chx10-neuron counts in the spinal cord was performed as previously described.^22^ Briefly, five cryosections of 20 μm thickness with a 320 μm interval were collected from the lumbar spinal cord (L3-L6 level) per animal, and the number of FoxP2(+) or Chx10(+) cells were counted for each hemicord section. For the quantification of CC3-stained neuronal counts and areas, a ROI of 625 μm × 625 μm field size was set for each brain region in the brain hemisphere. An Alexa 594-detected CC3 image and a DAPI-stained nucleus image on the same z-plane were taken for each brain region with the Dragonfly confocal laser microscope. Images were then processed with ImageJ 1.53 software for background subtraction and for thresholding the images. To count cell numbers, particles of the same size as nucleus that were double-positive for CC3 and DAPI staining were counted. To quantify CC3-positive areas, the total area of pixels with above-threshold intensity was measured. Particles corresponding to staining noise were excluded by setting the threshold for particle size. Statistical analysis was conducted using Prism 7.05 (GraphPad, San Diego, CA).

### Quantification and statistical analysis

Statistical analysis was conducted using Prism 7.05 (GraphPad, San Diego, CA). The data are expressed as the mean ± SD. For the analysis in Figures 2I and 2J, one-way analysis of variance (ANOVA) and Tukey’s *post-hoc* test was applied. For the analysis in Figures 4D and 4E, Mann–Whitney *U*–test was applied. p Values <0.05 were considered statistically significant. The details for each experiment including the number of animals are specified in the figure legends.

## Data Availability

• All data are available in the manuscript or supplemental information. • All codes are available in the supplemental information. • Any additional information required to reanalyze the data reported in this paper is available from the lead contact upon request.

## Supplemental information

Document S1. Figure S1 and Table S1

Video S3. Response of a neonatal mutant mouse harboring only a single allele of α- and β-clusters (αβ/-) to tail pinch, related to Figure 2

Video S4. Response of a neonatal mutant mouse harboring a single allele of α- and β-clusters and TC allele (αβ/TC) to tail pinch, related to Figure 2
