# Isoform requirement of clustered protocadherin for preventing neuronal apoptosis and neonatal lethality

**DOI:** 10.1016/j.isci.2024.109606

**Published:** 2024-04-01

**Authors:** Hiroaki Kobayashi, Kenji Takemoto, Makoto Sanbo, Masumi Hirabayashi, Takahiro Hirabayashi, Teruyoshi Hirayama, Hiroshi Kiyonari, Takaya Abe, Takeshi Yagi

## Main text

(iScience *26*, 105766; January 20, 2023)

In the initial publication of the article, an error occurred during the preparation and assembly of Figures 5B–5E. Specifically, while adjusting the size and position of the panels for the *in situ* hybridization (ISH), the micrograph of *Pcdha12* was mistakenly used twice, leading to identical micrographs in Figures 5B and 5C. This now has been corrected by replacing the original Figure 5C with the correct *Pccdhb22* ISH micrograph. This correction has been applied to both the online version and the PDF of the article. The adjustments were made after a thorough review of the raw data and an examination of the image analysis process, including any modifications to the images either for analytical purposes or during the preparation of the figures. This correction was conducted to ensure the transparency and reproducibility of the findings and was deemed necessary by the editorial team to maintain accurate reporting. The authors have verified these changes and confirm that they do not affect the scientific conclusions of the study. The authors apologize for any confusion and inconvenience caused to readers.

**Figure:**
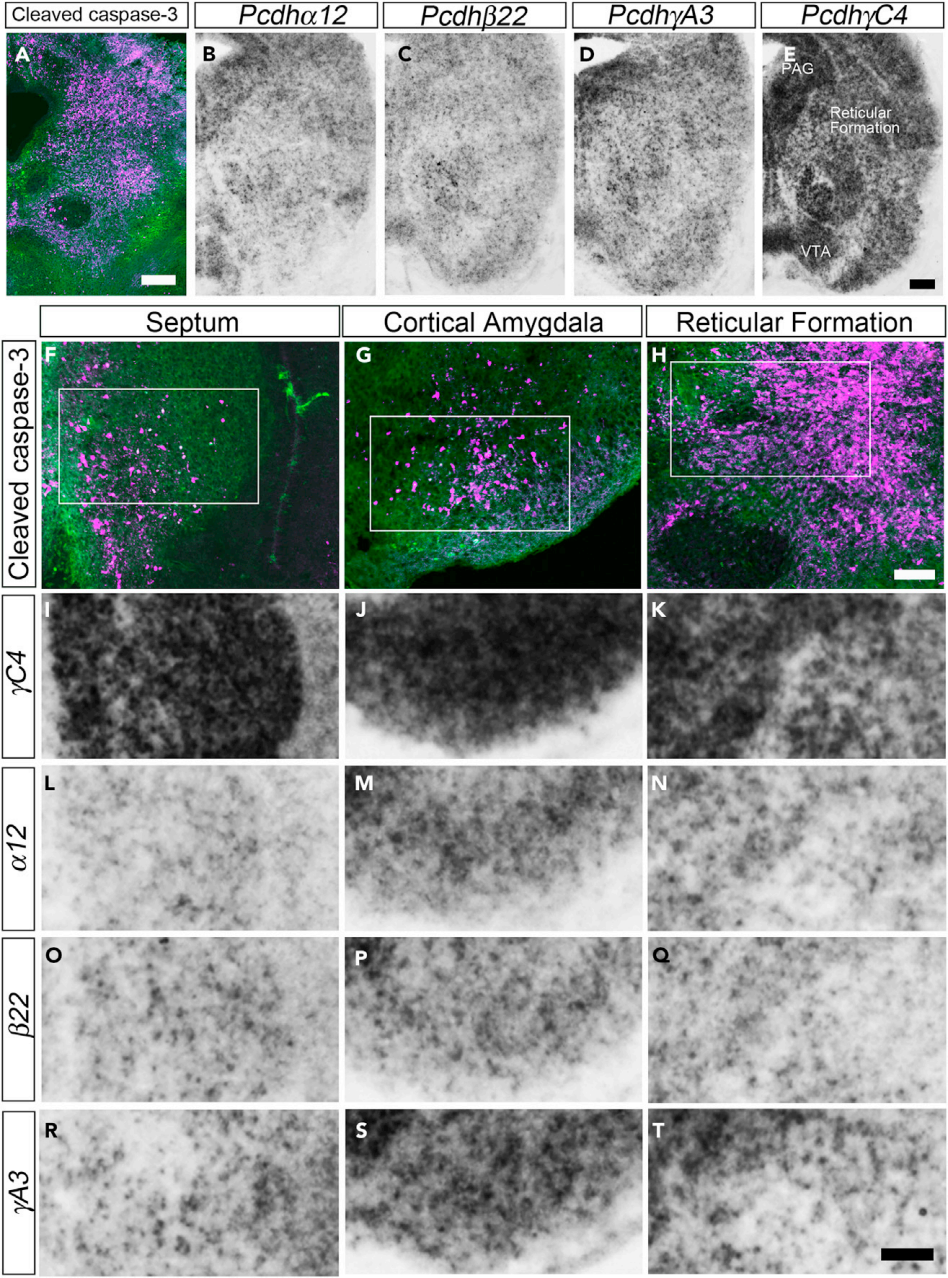
Figure 5. Nuclei susceptible to apoptosis in *TC* mutants exhibited the combinatorial expression of dominant *γC4* and stochastic isoforms (corrected)

