# Supplementary material for: Isoform requirement of clustered protocadherin for preventing neuronal apoptosis and neonatal lethality
Source: iScience. 2022 Dec 8;26(1):105766. doi: 10.1016/j.isci.2022.105766 (PMC9793319; doi:10.1016/j.isci.2022.105766)
Supplement: Document S1. Figure S1 and Table S1 [file mmc1.pdf]

iScience, Volume *26*

Supplemental information

Isoform requirement of clustered protocadherin for preventing neuronal apoptosis and neonatal lethality

Hiroaki Kobayashi, Kenji Takemoto, Makoto Sanbo, Masumi Hirabayashi, Takahiro Hirabayashi, Teruyoshi Hirayama, Hiroshi Kiyonari, Takaya Abe, and Takeshi Yagi

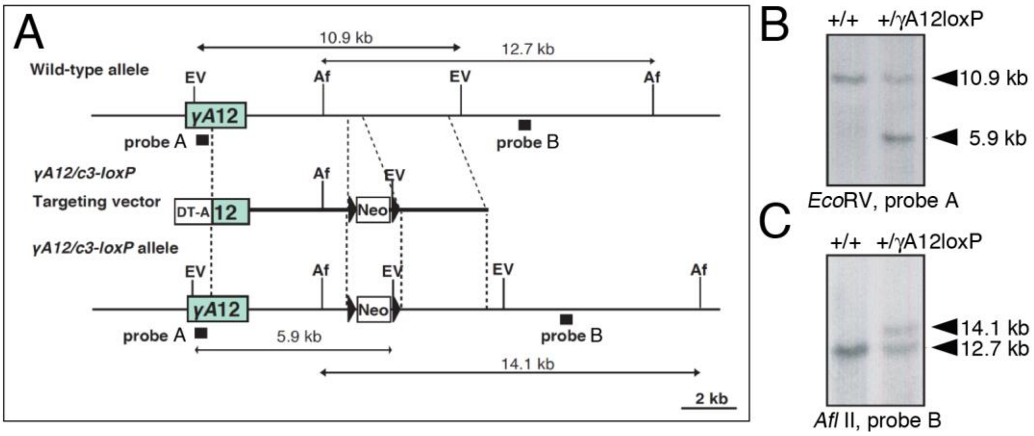

Figure S1. Design of a *γA12/C3-loxP* targeting construct, related to Figure 1.
(A) Diagram showing the *γA12/C3-loxP* targeting constructs. EV: EcoRV, Af: AflII. Filled triangles represent *loxP* sites. (B) Southern blot of homologous recombinant ES cells digested by EcoRV, with probe A. (C) Southern blot of homologous recombinant ES cells digested by AflII, with Probe B.

Table S1. List of oligonucleotides, related to STAR Methods.

Probe sequences used for southern blot.

Name	Sequence: (+)-strand
Probe A	CAGCGAAAGTTTTCAAGCTGGACAGTAACTTGGGGACAATATCAACAATAG GGGAGCTGAATCATGAAGAATCGGGGTCGAACGACAACGCACCTGCGTTT GCTCGGTCCGAGTACCGCGTGAGCGTGCGGGAGAACGTGGCTGTGGGCA CTCAGCTGCTGCTGGTCAACGCCACCGACCCAGACGAAGGCGCCAATGCG GAAGTGATCTATTCCTTCCGGTACGTGGACGACAAGGTCTACGAGATGGAG GTGCAAGCTACAGATAACGCGGGATATTCTGCACGGGCCAAGGTCCTGGT CACAGTTC
Probe B	CCTGTGAAACAGCTCCGTCATGACAGCTAGCTTCCATCAGACCAAGCAAAG GAGGGAGCCAGAGAGGGAAAATCTGATAAATGCTTCAATGCTTATGTGAGC CCCAATTTGAAGTGACACGCGTTACTACAAATATATTCAGATCATTACAAAC AAAATCATTTGCTCCTGCCCACACTCAGAAGAGATGAATTTCAGTCCACTTA TGATGGTTTTCTGTGTTCTGAGAGGAAACTCCCCTTCTCTTGTTTCTATTCAT GTATGGTCGTGGGTCGTGCATCTGTTCAGTAAGATTATTATTCCATTACTGC TGTATTTTATTTTGATCTCAAACTGCTCTAGATT

Primers used for genotyping the mutant mice

Name	Sequence	Purpose
α1-232F (F1)	TGCAGACCATTGCTGGATGT	Pcdh (common)
gA12C3 intron 4846R (R2)	GCACGTTCAGGGCGTACATA	Pcdh (TC allele)
Pcdhα1R1 (R1)	CAATGTCTGCGTCCACGGCG	Pcdh (WT allele)
115F	GCGCGCCAAAGCTTGCATGC	Taf7-tg
115R	CTCTCCCTATAGTGAGTC	Taf7-tg

Primers used for RT-PCR/real-time RT-PCR

Name	Sequence	Purpose
CR2-3′F	CAGTGGCCAACAGTATCCAG	Pcdhα CR
C3-5′	AGGTCCAGCTGTTGCTGTTG	Pcdhα CR
Pcdhb3 real F	CTCTCTTGGTCCTGGTAAACTTC	Pcdhβ3
Pcdhb3 real R	GGGATACCATGGCTTGAATTACTG	Pcdhβ3
Pcdhb21 real F	AAAGCCTGTCTCCCAACCTTG	Pcdhβ21
Pcdhb21 real R	CAGGAACCATGATGAGAGTCAAC	Pcdhβ21
PcdhgA3 real F	CTCAAGATTTACTTGAAACGAAAGAAGACC	PcdhγA3
PcdhgB7 real F	GGCACTGTTGGCTAGTATTTTAACTC	PcdhγB7
PcdhgC3 real F	CCTGTGTTCTATAGACAGGTGTTG	PcdhγC3
PcdhgC4 real F	GTCCACCCTCTGATCTTCTC	PcdhγC4
PcdhgC5 real F	GTTCCCGCTCTAGTACGCTG	PcdhγC5
Pcdhg constant R3	CAGGTGCCAGTTTCATCACC	Pcdhγ (common)
Gamma cp1 real F	CTGGCGTTTCTCTCAAGCCC	Pcdhγ CR
Pcdhg constant R2	CATGGCTTGCAGCATCTCTG	Pcdhγ CR
GapdhF3 770-789bp	AATGTGTCCGTCGTGGATCT	GAPDH
GapdhR3 897-916bp	GTTGAAGTCGCAGGAGACAA	GAPDH

Primers used for *in situ* hybridization RNA probes

Name	Sequence	Purpose
Pcdhα12 forward	CGGGATCCCCTGAAACCAACAAAAAC	Pcdhα12
Pcdhα12 reverse	CCGCTCGAGGACCTCTGGTGCATTATCA	Pcdhα12
Pcdhβ22 forward	AAGCTTCGTTGACATCCACCACCTG	Pcdhβ22
Pcdhβ22 reverse	GGGCCCCGCCGCTACTTTGTGATGG	Pcdhβ22
PcdhγA3 forward	TAGTGGTACCTGTCTCGTCAGCTTTAACATCCTTGTG	PcdhγA3
PcdhγA3 reverse	TAGTGAGCTCCCCATCCGTGGCTCTTAGAC	PcdhγA3
PcdhγC3 forward	AACAATCCCTCTTTCCCCACCGG	PcdhγC3
PcdhγC3 reverse	ATGCGCTCCTTCGGGATTGG	PcdhγC3
PcdhγC4 forward	TAGTGGTACCAGGCTGCAGGTCGCTGG	PcdhγC4
PcdhγC4 reverse	TAGTACTAGTTGGTAAGTGGTCGGGAATCCG	PcdhγC4
